# Supplementary material for: The Systems Biology Research Tool: evolvable open-source software
Source: BMC Syst Biol. 2008 Jun 29;2:55. doi: 10.1186/1752-0509-2-55 (PMC2446383; doi:10.1186/1752-0509-2-55)
Supplement: Additional file 1 — SBRT Archive. An archive of the current version of the Systems Biology Research Tool. [file 1752-0509-2-55-S1.zip › sbrt-1.4.0/doc/developers_guide/api/sbrt/ext/ExternalSoftwareException.html]

ExternalSoftwareException


|  |  |  |
| --- | --- | --- |
| **Overview** \| **Package** \| **Class** \| **Use** \| **Tree** \| **Deprecated** \| **Index** \| **Help** | |  |
| PREV CLASS   NEXT CLASS | **FRAMES**    **NO FRAMES**     **All Classes** |  |
| SUMMARY: NESTED \| FIELD \| CONSTR \| METHOD | DETAIL: FIELD \| CONSTR \| METHOD |  |


---


## sbrt.ext Class ExternalSoftwareException

```
java.lang.Object
  java.lang.Throwable
      java.lang.Exception
          java.lang.RuntimeException
              sbrt.ext.ExternalSoftwareException
```

**All Implemented Interfaces:**: java.io.Serializable

**Direct Known Subclasses:**: ProgramSolverException

---

``` public class ExternalSoftwareException extends java.lang.RuntimeException ```

This class is used to wrap exceptions thrown by external software packages.
If an exception is thrown by an external software package, and there is no
reasonable way to handle it, it should be caught, wrapped in an instance of
this class, and this new object thrown.

**Author:**
:   This class was written and documented by
    Jeremiah Wright while in the Wagner lab.

**See Also:**: Serialized Form

---

**Constructor Summary**

`ExternalSoftwareException(java.lang.String message,
java.lang.Throwable cause)`
  
          Constructs an external software exception.

`ExternalSoftwareException(java.lang.Throwable cause)`
  
          Constructs an external software exception.


**Method Summary**

**Methods inherited from class java.lang.Throwable**

`fillInStackTrace, getCause, getLocalizedMessage, getMessage, getStackTrace, initCause, printStackTrace, printStackTrace, printStackTrace, setStackTrace, toString`

**Methods inherited from class java.lang.Object**

`clone, equals, finalize, getClass, hashCode, notify, notifyAll, wait, wait, wait`

**Constructor Detail**

### ExternalSoftwareException

```
public ExternalSoftwareException(java.lang.Throwable cause)
```

:   Constructs an external software exception.

    **Parameters:**: `cause` - the original exception throw by the external software package.

---


### ExternalSoftwareException

```
public ExternalSoftwareException(java.lang.String message,
                                 java.lang.Throwable cause)
```

:   Constructs an external software exception.

    **Parameters:**: `message` - a descriptive message useful for debugging.: `cause` - the original exception throw by the external software package.


---


|  |  |  |
| --- | --- | --- |
| **Overview** \| **Package** \| **Class** \| **Use** \| **Tree** \| **Deprecated** \| **Index** \| **Help** | |  |
| PREV CLASS   NEXT CLASS | **FRAMES**    **NO FRAMES**     **All Classes** |  |
| SUMMARY: NESTED \| FIELD \| CONSTR \| METHOD | DETAIL: FIELD \| CONSTR \| METHOD |  |


---
